# The complete chloroplast genome of *Akebia quinata* (Lardizabalaceae) and phylogenetic analysis

**DOI:** 10.1080/23802359.2020.1797557

**Published:** 2020-07-27

**Authors:** Jie Min, Tao Tao

**Affiliations:** The Third Hospital of Nanchang, Nanchang, China

**Keywords:** *Akebia quinata*, Lardizabalaceae, phylogenetic analysis, chloroplast, genome sequence

## Abstract

*Akebia quinata* is an herb medicine plant in traditional oriental medicine in China. In this current study, we had assembled the complete chloroplast genome of *A. quinata.* The complete chloroplast genome sequence of *A. quinata* has a total 157,817 bp in size and has a typical quadripartite structure, which is the same as other plant species chloroplast genome. It contained a large single-copy (LSC) region of 86,544 bp, a small single-copy (SSC) region of 18,989 bp and two inverted repeat (IR) regions of 26,142 bp. The complete chloroplast genome sequence contains 132 genes, including 87 encoding genes, 37 transfer RNA genes, and eight ribosomal RNA genes. One of IR region harbors seven protein encoding genes, seven tRNA genes, and four rRNA genes. Phylogenetic analysis result showed that *A. quinata* is closely related to *Decaisnea insignis* of the family Lardizabalaceae using the maximum-likelihood (ML) method in this study.

*Akebia quinata* is an herb medicine plant in traditional oriental medicine in China. *Akebia quinata* (Lardizabalaceae) is a creeping woody vine that is widely distributed in East Asia, including China, Korea, and Japan (Sun et al. 2018). *Akebia quinata* is the cultivation of woods, hedges, and thickets in mountainous areas, but also is at forest margins along streams, scrub on mountain slopes at elevations of 300–1500 m in China. *Akebia quinata* has long time been widely used as an antiphlogistic, a diuretic, and an analgesic in many East Asia countries (Kitaoka et al. 2009). *Akebia quinata* is one of the important herbal medicines in China, but fewer genomic information and study has been reported of this species. Over here, we had assembled the complete chloroplast genome of *A. quinata* with the good knowledge of chloroplast genome information of this species that can provide the traditional Chinese medicine study and utilization in the future.

The chloroplast DNA of *A quinata* was extracted from its fresh stems which were sampled in the market of herb near the Third Hospital of Nanchang (28.66 N, 115.90E). The corresponding voucher herbarium specimen was stored at the Third Hospital of Nanchang and the accession number was THNC-03. We controlled and removed the sequences using FastQC (Andrews 2015) after which the chloroplast genome DNA was purified and sequenced. The chloroplast genome was constructed with de novo assembly and mapped using NOVOPlasty (Dierckxsens et al. 2017). The chloroplast genome of *A. quinata* gene annotation was done using Geneious (Kearse et al. 2012). All the genes were analyzed using CPGAVAS (Liu et al. 2012) and combined with the online alignment tool Blastx and ORF Finder from the NCBI web (http://www.ncbi.nlm.nih.gov/). The tRNA genes were predicted using the online sites tRNA-scan (Lowe and Chan 2016). The circular chloroplast genome map was finished using OrganellarGenomeDRAW (Greiner et al. 2019) (http://ogdraw.mpimp-golm.mpg.de/).

The chloroplast genome sequence of *A. quinata* had been submitted to NCBI and the accession number was NK9912251. The complete chloroplast genome sequence of *A. quinata* has a total 157,817 bp in size and has a typical quadripartite structure, which is the same as other plant species chloroplast genome. It contained a large single-copy (LSC) region of 86,544 bp, a small single-copy (SSC) region of 18,989 bp and two inverted repeat (IR) regions of 26,142 bp. The overall nucleotide composition of chloroplast genome sequence is A 30.3% (47,841 bp), T 31.0% (48,952 bp), C 19.7% (31,052 bp), G 19.0% (29,972 bp), and the total G + C content of 38.7%. The complete chloroplast genome sequence contains 132 genes, including 87 encoding genes, 37 transfer RNA genes, and eight ribosomal RNA genes. One of IR region harbors seven protein encoding genes (rpl2, rpl23, ycf2, ndhB, rps7, rps12, and ycf1), seven tRNA genes (trnI-CAU, trnL-CAA, trnV-GAC, trnIGAU, trnA-UGC, trnR-ACG, and trnN-GUU), and four rRNA genes (rrn16, rrn23, rrn4.5, and rrn5).

The phylogenetic analyses were carried out using the complete chloroplast genome sequences of *A. quinata* and 12 plant species reported the complete chloroplast genome. Before constructing the phylogenetic tree, sequence alignment of all the species was performed with MEGA X (Kumar et al. 2018). The maximum-likelihood (ML) phylogenetic tree was constructed using MEGA X. The ML phylogenetic tree was inferred with strong support and used the bootstrap values from 2000 replicates at all the nodes. The ML tree was drawn and edited using MEGA X. Phylogenetic analysis result (Figure 1) showed that *A. quinata* is closely related to *Decaisnea insignis* of the family Lardizabalaceae using the ML method in this study. The good knowledge of chloroplast genome information of this species can provide the traditional Chinese medicine study and utilization in the future.

**Figure 1.**
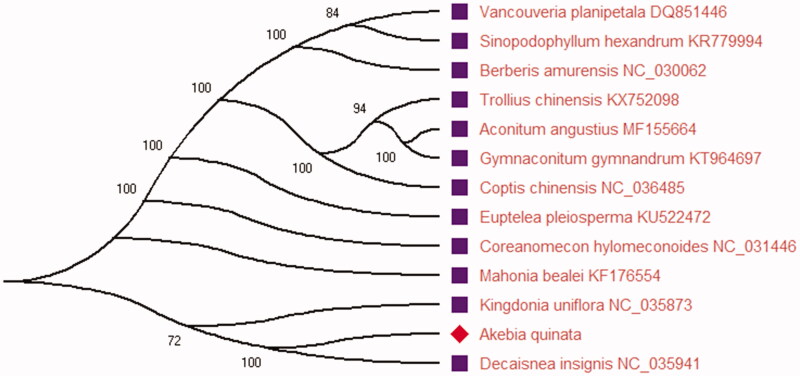
The phylogenetic ML tree constructed using MEGA X based on the complete chloroplast sequence of 13 plant species. Bootstrap support values are shown next to all the nodes based on 2000 replicates. The accession numbers are in the figure.

## Data Availability

The data that support the findings of this study are available from the corresponding author, upon reasonable request.

## Data openly available in a public repository that issues datasets with DOIs

The data that support the findings of this study are openly available in *Akebia quinata* at http://doi.org/10.1080/23802359.2020.1797557, reference number [NK9912251].

## References

[CIT0001] Andrews S. 2015. FastQC: a quality control tool for high throughput sequence data. http://www.bioinformatics.babraham.ac.uk/projects/fastqc/.

[CIT0002] Dierckxsens N, Mardulyn P, Smits G. 2017. NOVOPlasty: de novo assembly of organelle genomes from whole genome data. Nucleic Acids Res. 45(4):e18. 28204566 10.1093/nar/gkw955 PMC5389512

[CIT0003] Greiner S, Lehwark P, Bock R. 2019. OrganellarGenomeDRAW (OGDRAW) version 1.3.1: expanded toolkit for the graphical visualization of organellar genomes. Nucleic Acids Res. 47(W1):W59–W64. 30949694 10.1093/nar/gkz238 PMC6602502

[CIT0004] Kearse M, Moir R, Wilson A, Stones-Havas S, Cheung M, Sturrock S, Buxton S, Cooper A, Markowitz S, Duran C, et al. 2012. Geneious basic: an integrated and extendable desktop software platform for the organization and analysis of sequence data. Bioinformatics. 28(12):1647–1649. 22543367 10.1093/bioinformatics/bts199 PMC3371832

[CIT0005] Kitaoka F, Kakiuchi N, Long CF, Itoga M, Mitsue A, Mouri C, Mikage M. 2009. Molecular characterization of Akebia plants and the derived traditional herbal medicine. Biol Pharm Bull. 32(4):665–670. 19336902 10.1248/bpb.32.665

[CIT0006] Kumar S, Stecher G, Li M, Knyaz C, Tamura K. 2018. MEGA X: molecular evolutionary genetics analysis across computing platforms. Mol Biol Evol. 35(6):1547–1549. 29722887 10.1093/molbev/msy096 PMC5967553

[CIT0007] Liu C, Shi L, Zhu Y, Chen H, Zhang J, Lin X, Guan X. 2012. CpGAVAS, an integrated web server for the annotation, visualization, analysis, and GenBank submission of completely sequenced chloroplast genome sequences. BMC Genomics. 13:715. 23256920 10.1186/1471-2164-13-715 PMC3543216

[CIT0008] Lowe TM, Chan PP. 2016. tRNAscan-SE On-line: integrating search and context for analysis of transfer RNA genes. Nucleic Acids Res. 44(W1):W54–W57. 27174935 10.1093/nar/gkw413 PMC4987944

[CIT0009] Sun HP, Seol J, Lee SW, Sun DP, Sung YY, Kim HK. 2018. *Akebia quinata* decaisne aqueous extract acts as a novel anti fatigue agent in mice exposed to chronic restraint stress. J Ethnopharmacol. 222:270–279. 29630998 10.1016/j.jep.2018.04.010

